# High‐intensity interval exercise and glycemic control in adolescents with type one diabetes mellitus: a case study

**DOI:** 10.14814/phy2.13339

**Published:** 2017-07-12

**Authors:** Emma J. Cockcroft, Christopher Moudiotis, Julie Kitchen, Bert Bond, Craig A. Williams, Alan R. Barker

**Affiliations:** ^1^ Children's Health and Exercise Research Centre Sport and Health Sciences College of Life and Environmental Sciences University of Exeter Exeter UK; ^2^ Royal Devon and Exeter NHS Foundation Trust Exeter UK

**Keywords:** Cardiovascular disease, case report, hypoglycemia, insulin, youth

## Abstract

Current physical activity guidelines for youth with type 1 diabetes (T1D) are poorly supported by empirical evidence and the optimal dose of physical activity to improve glycemic control is unknown. This case report documents the effect of acute high‐intensity interval exercise (HIIE) and moderate‐intensity exercise (MIE) on 24‐h glycemic control in three adolescents with T1D using continuous glucose monitoring. Results highlight varied individual response to exercise across the participants. In two participants both MIE and HIIE resulted in a drop in blood glucose during exercise (−38 to −42% for MIE and −21–46% in HIIE) and in one participant both MIE and HIIE resulted in increased blood glucose (+19% and + 36%, respectively). Over the 24‐h period average blood glucose was lower for all participants in the HIIE condition, and for two for the MIE condition, compared to no exercise. All three participants reported HIIE to be more enjoyable than MIE. These data show both HIIE and MIE have the potential to improve short‐term glycemic control in youth with T1D but HIIE was more enjoyable. Future work with a larger sample size is required to explore the potential for HIIE to improve health markers in youth with T1D.

## Introduction

Cardiovascular disease (CVD) is a major cause of mortality in adults with type one diabetes mellitus (T1D) (Soedamah‐Muthu et al. 2006), and is related to glycemic control as measured using glycated hemoglobin (HbA1c) (Juutilainen et al. 2008). Subclinical signs of CVD and clustering of CVD risk factors are, however, present in children with T1D (Snell‐Bergeon and Nadeau 2012). It is therefore important to identify interventions which can reduce CVD risk and improve glycemic control in youth with T1D.

The therapeutic effects of physical activity in the management of CVD risk in youth with T1D are established (Mosso et al. 2015). Consequently, the American Diabetes Association recommend children and adolescents with T1D to undertake at least 30–60 min of daily moderate to vigorous physical activity (MVPA) (Silverstein et al. 2005). However, the optimum exercise recommendations for youth with T1D are unknown, especially for improving glycemic control. In healthy adolescents a single bout of time efficient high‐intensity interval exercise (HIIE) has been shown to improve glucose tolerance and insulin sensitivity (Cockcroft et al. 2015), suggesting that HIIE may be a strategy to manage glycemic control in youth with T1D. While an acute bout of HIIE has been shown to improve postprandial and 24‐h glycemic control in adults with type two diabetes mellitus (Gillen et al. 2012), no data currently exist in youth with T1D.

The purpose of this case study is to document across three patients with T1D varying in sex, baseline glycemic control and aerobic fitness, the acute effect of HIIE on glycemic control during exercise, in response to a meal challenge and over a 24‐h period and compare this to 30 min of moderate intensity exercise (MIE).

## Patient Information

This case report presents data on the three participants who were originally recruited to a study, which closed due to inadequate recruitment, examining exercise and glycemic control in youth with T1D. The participants consisted of one female (participant A: 17.1 years) and two males (participant B: 14.8 years, and participant C: 16.6 years) with T1D of at least 3 years duration (see Table 1 for participant characteristics). All participants were on a basal‐bolus insulin regime. Informed parental consent and participant assent were obtained and ethical approval was granted by the National Health Service Research Ethics Committee (14/SW/1028).

**Table 1:** Participants descriptive characteristics

	Participant A	Participant B	Participant C
Age, years	17.1	14.8	16.6
Sex	Female	Male	Male
HbA1c (mmol/mol)	62	59	37
Body mass, kg	77.6	50.4	61.8
Stature, m	1.62	1.72	1.78
Body fat, %	18.8	23.0	7.6
Peak power, W	259	173	442
$\dot{V}$O_2_ max (L·min^−1^)	2.69	1.84	3.63
$\dot{V}$O_2_ max (mL·kg·min^−1^)	34.6	36.5	58.7
GET (L·min^−1^)	1.76	1.01	2.29
GET (%$\dot{V}$O_2_ max)	65%	55%	63%

Results shown as individual values. HbA1c, Glycated hemoglobin.

### Experimental design

Participants attended the laboratory on four separate occasions, consisting of a preliminary baseline assessment visit and three experimental conditions. On the baseline assessment visit, stature, body mass and body composition (BodPod^®^, COSMED) were measured before participants undertook a combined ramp‐incremental and supramaximal test to exhaustion to determine peak power, maximal oxygen uptake ($\dot{V}$O_2_ max) and the gas exchange threshold (GET) (Barker et al. 2011).

Each experimental condition consisted of 4 days of data collection. On day 1, participants were fitted with a continuous glucose monitoring system (CGMS) (iPro 2, Medtronic, USA) and provided with a food intake and insulin administration diary. On day 2, participants continued to wear the CGMS. On day 3, participants attended the laboratory at 08:00 following an overnight fast where they completed an exercise intervention (HIIE, MIE or a non‐exercise control) and test meal, as described below. On the afternoon of day 4 the CGMS was removed. Participants were not tested on day 3 if they had experienced a hypoglycemic episode in the preceding 24 h.

### Experimental intervention day protocol

At 08:30 participants consumed a standardized breakfast (64 g carbohydrate (CHO), 17 g protein, 8 g of fat, 412 kcal of energy) and rested in the laboratory. At 10:30 participants undertook one of the following conditions in a counterbalanced order: (1) HIIE: 3 min warm up at 20 W followed by eight bouts of 1 min cycling at 90% of peak power interspersed with 1.25 min recovery at 20 W followed by a 3 min cool down at 20 W; (2) MIE: continuous cycling for 30 min at 90% GET; and (3) rest in the laboratory (CON). Rating of perceived exertion (RPE) was taken every 5 min during MIE and after each interval in HIIE. Following exercise, participants completed the Physical Activity Enjoyment Scale (PACES) (Motl et al. 2001). At 12:00 a mixed meal tolerance test (MMTT) was undertaken where participants consumed a liquid meal (Ensure Plus High Protein, 6 mL per kg (maximum 360 mL), content per 100 mL: CHO 15.9 g, energy 125 kcal) (Oram et al. 2014). Participants remained in the laboratory over the 4‐h postprandial period. Participants were advised to manage glucose levels as normal throughout each experimental condition, and to record insulin dose, and treatment of hypoglycemia in the diary provided. All participants administered their insulin 10–15 min pre‐meal (e.g., breakfast, MMTT) during all experimental visits.

### Data analyses

Data are reported for each participant using descriptive statistics (e.g., mean ± SD). Food diaries were assessed for total energy and CHO intake (Nutritics, Nutritics LTD, Ireland). Mean blood glucose and time spent in hyper‐ (>7.2 mmol L^−1^), eu‐ (3.9–7.2 mmol L^−1^), and hypo‐ (<3.9 mmol L^−1^) glycemia were assessed over a 24‐h period (08:00 on day 3 to 08:00 on day 4), and during exercise, following the MMTT and the night after exercise (23:00–06:00) using the CGMS (Clarke and Kovatchev 2009). Dietary CHO intake and insulin use were used to calculate the insulin: CHO ratio.

## Outcomes

### Cardiorespiratory and enjoyment responses to exercise

Mean $\dot{V}$O_2_ was 1.70 ± 0.60 L min^−1^ and 1.99 ± 0.98 L min^−1^ for HIIE and MIE, respectively, but peak $\dot{V}$O_2_ during HIIE attained 2.86 ± 1.04 L min^−1^ (98% of $\dot{V}$O_2_ max). Average RPE was higher in HIIE compared to MIE (8 ± 1 vs. 6 ± 1) and all participants found HIIE more enjoyable than MIE (PACES: HIIE: 68 ± 1 vs. MIE: 57 ± 3).

### Glycemic response to exercise

**Figure 1:**
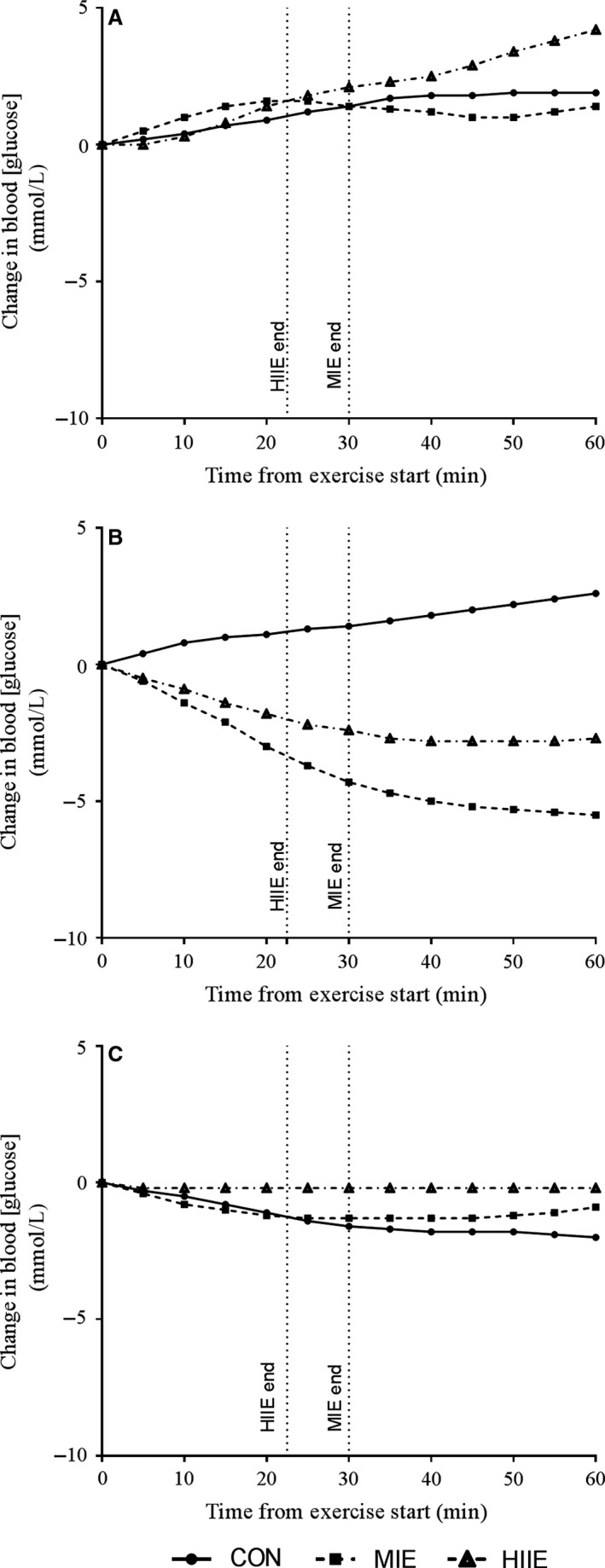
Individual glycemic response to exercise (participants A, B and C). Moderate‐intensity exercise (MIE), high‐intensity interval exercise (HIIE) and rest (CON). Exercise was performed 2 h after breakfast. Blood glucose levels at 0 min for CON, MIE and HIIE, respectively, were: Participant A; 8.1 mmol/L, 7.5 mmol/L and 5.8 mmol/L; Particpants B: 5.3 mmol/L, 12.4 mmol/L 5.6 mmol/L; and Participant C: 7.7 mmol/L, 4.4 mmol/L and 3.3 mmol/L.

As shown in Figure 1, for participants B and C, both MIE and HIIE coincided with a drop in blood glucose from pre to post exercise (Participant B: MIE: −35% (−4.3 mmol L^−1^), HIIE: −43% (−2.4 mmol L^−1^); Participant C: MIE: −35% (−1.3 mmol L^−1^), HIIE: −8% (−0.2 mmol L^−1^). In participant A, blood glucose rose in HIIE (+36%, +2.1 mmol L^−1^) and MIE (+19%, +1.4 mmol L^−1^).

### 24‐h, MMTT and nocturnal glycemic responses

Blood glucose data during the MMTT, 24‐h post exercise period and the night after exercise are presented in Table 2.

**Table 2:** The effects of acute MIE and HIIE on 24‐h glycemic control, postprandial response to MMTT and overnight glycemia in three adolescents with T1D

	Participant A			Participant B			Participant C		
	CON	MIE	HIE	CON	MIE	HIIE	CON	MIE	HIIE
24‐h									
Total 24‐h bolus insulin (units)	32	28	19	24	15	17	32	28	19
Mean blood [glucose], mmol L^−1^	10.8	10.9	7.2	7.2	8.5	6.7	5.9	5.5	4.2
% hyperglycemia	87%	91%	73%	46%	51%	47%	20%	12%	9%
% euglycemia	13%	6%	12%	14%	28%	37%	79%	71%	34%
% hypoglycemia	0%	3%	15%	40%	21%	16%	2%	16%	57%
Hypoglycemic events	–	1	2	2	2	2	1	3	5
MMTT									
Insulin units with meal	7	7.5	6	4	0	0	9.5	9	0
Mean blood [glucose], mmol L^−1^	11.3	8.5	7.9	7.5	9.6	8.4	5.7	4.1	3.2
tAUC [glucose]	1360	1026	956	890	1154	1013	690	500	393
% hyperglycemia	100%	56%	48%	48%	76%	76%	20%	0%	0%
% euglycemia	0%	36%	32%	52%	24%	24%	80%	56%	28%
% hypoglycemia	0%	0%	20%	0%	0%	0%	0%	44%	72%
Hypoglycemic event	–	✓	✓	–	–	–	–	✓	✓
Nocturnal									
Mean blood [glucose], mmol L^−1^	11.3	8.5	7.9	7.5	9.6	8.4	5.7	4.1	3.2
% hyperglycemia	100%	100%	40%	31%	0%	47%	0%	8%	0%
% euglycemia	0%	0%	13%	9%	44%	53%	100%	85%	13%
% hypoglycemia	0%	0%	35%	60%	56%	0%	0%	7%	87%
Hypoglycemic event	–	–	✓	✓	✓	–	–	✓	✓

Results shown as individual values. 4‐h tAUC; Total area under curve, MMTT; mixed meal tolerance test. % represents percentage of total time spent in hyper‐ (>7.2 mmol L^−1^), eu‐ (3.9–7.2 mmol L^−1^), and hypo‐ (<3.9 mmol L^−1^) glycemia.

### Carbohydrate and insulin

Mean CHO intake for the day prior to the laboratory visit (CON: 263 ± 42 g, MIE: 267 ± 50 g and HIIE: 269 ± 28 g), the day of the laboratory visit (CON: 278 ± 63 g, MIE: 269 ± 54 g and HIIE: 278 ± 28 g), and the morning post the laboratory visit (CON: 93 ± 11 g, MIE: 80 ± 11 g and HIIE: 82 ± 4 g) were similar across conditions. Mean bolus insulin for the day prior to the laboratory visit (participant A: 23, 25 and 25 units; participant B: 25, 22, and 16 units; participant C: 12, 15 and 9 units for CON, MIE and HIIE, respectively) was also similar across conditions. Insulin dose for each laboratory visit day is shown in Table 2. The insulin: CHO ratio for CON, MIE, HIIE were: Participant A: 9, 12 and 14; Participant B: 10, 14 and 16; and Participant C: 12, 12 and 13.

## Discussion

This study provides insight into changes in glycemic control over a 24‐h period after an acute bout of HIIE and MIE in three adolescents with T1D. The data highlight the potential of HIIE to improve 24‐h glycemic control and postprandial hyperglycemia in adolescents with T1D, and that they found this form of exercise more enjoyable than MIE.

Our results show reduced 24‐h glucose levels in all participants for HIIE compared to CON and for two patients for MIE compared to CON, which was partially due to the reduced average postprandial glucose assessed during a MMTT. These findings support previous research in adults with type two diabetes (Gillen et al. 2012), where a similar HIIE protocol (10 × 1 min at 90% maximal heart rate), lowered average postprandial glucose and time spent in hyperglycemia over 24‐h. We also observed a reduced time spent in postprandial hyperglycemia following HIIE, which may have important clinical implications given its association with disease development (Ceriello 2005).

This study highlights glucose perturbations during HIIE and MIE in adolescents with T1D. Blood glucose fell by 38–42% during MIE and 21–46% during HIIE in two participants. These findings concur with previous work by Tsalikian et al. (2005) who reported that during 60 min of MIE, 82% of participants experienced at least a 25% decrease in glucose compared to pre‐exercise. Conversely, previous research in adults with T1D showed less of a decline in blood glucose following sprint interval exercise, compared to MIE (Guelfi et al. 2005). This is contradictory to the present study where the drop in blood glucose during HIIE was more pronounced than MIE. This disparity may be due to the “all‐out” nature of the sprint interval exercise in the study by Guelfi et al. (2005) which is known to increased hepatic glucose output (Riddell and Perkins 2009). It is important to note the different response of participant A compared to participants B and C showing an increase in glucose during exercise. This could be due to a number of factors, including differences in sex (Horton et al. 2006), maturation status (Riddell 2008) and physical fitness (Mosso et al. 2015), with participant A being female as well as the oldest and least fit participant.

Results from this study highlight nocturnal hypoglycemia following exercise. In two participants, HIIE was associated with an increase in time spent in nocturnal hypoglycemia (35% and 87% compared to 0% in CON), whereas MIE was associated with nocturnal hypoglycemia in one participant (7% compared to 0%). This increased incidence of nocturnal hypoglycemia has been demonstrated previously in children with T1D (aged 11–17 years), with 22% of participants experiencing hypoglycemia the night following 60 min of afternoon MIE (Tsalikian et al. 2005). The risk of nocturnal hypoglycemia after HIIE in youth with T1D is poorly understood, but is likely due to HIIE having greater insulin sensitizing effects than MIE (Cockcroft et al. 2015). This is indirectly supported in the present study by an increase in the insulin:CHO ratio in both exercise conditions but not CON, with a larger increase after HIIE. It is also noteworthy that the highest incidence of hypoglycemic events throughout the 24‐h period occurred in participant C, whose HbA_1c_ was the lowest of the participants. The observed hypoglycemic events after exercise may therefore, in part, be due to the low baseline glycemia in participant C.

In this study, participants found HIIE to be more enjoyable than MIE, which may have implications for implementing this type of exercise into an exercise intervention. Additionally, this study highlights large inter‐participant variability in the short‐term glycemic response to acute HIIE and MIE, indicative of the need to personalize glucose management with respect to modifying insulin dose and CHO intake before and after exercise. Despite the obvious limitation of a small samples size, data from this case study highlights HIIE as a potential target for future work in youth with T1D.

## Conflicts of Interest

None declared.
